# Risk Factors and Prognosis of Early Recurrence in Stage I–II Endometrial Cancer: A Large-Scale, Multi-Center, and Retrospective Study

**DOI:** 10.3389/fmed.2022.808037

**Published:** 2022-04-14

**Authors:** Yingyu Dou, Kun Song, Yu Fu, Yuanming Shen, Chuyao Zhang, Shuzhong Yao, Congjian Xu, Min Xia, Ge Lou, Jihong Liu, Bei Lin, Jianliu Wang, Weidong Zhao, Jieqing Zhang, Wenjun Cheng, Hongyan Guo, Ruixia Guo, Fengxia Xue, Xipeng Wang, Lili Han, Xia Zhao, Xiaomao Li, Ping Zhang, Jianguo Zhao, Jiezhi Ma, Wenting Li, Xiaohang Yang, Zizhuo Wang, Jingbo Liu, Yong Fang, Kezhen Li, Gang Chen, Chaoyang Sun, Xiaodong Cheng, Jie Jiang, Beibei Wang, Danfeng Luo, Beihua Kong, The Chinese Endometrial Carcinoma Consortium (CECC)

**Affiliations:** ^1^Cancer Biology Research Center (Key Laboratory of the Ministry of Education), Tongji Medical College, Tongji Hospital, Huazhong University of Science and Technology, Wuhan, China; ^2^Department of Gynecology and Obstetrics, Tongji Hospital, Tongji Medical College, Huazhong University of Science and Technology, Wuhan, China; ^3^Department of Obstetrics and Gynecology, Qilu Hospital, Cheeloo College of Medicine, Shandong University, Jinan, China; ^4^Women's Hospital, School of Medicine, Zhejiang University, Hangzhou, China; ^5^Department of Gynecologic Oncology, Sun Yat-sen University Cancer Center, Guangzhou, China; ^6^Department of Obstetrics and Gynecology, The First Affiliated Hospital of Sun Yat-sen University, Guangzhou, China; ^7^Department of Gynecology, Obstetrics and Gynecology Hospital of Fudan University, Shanghai, China; ^8^Department of Gynecology and Obstetrics, The Affiliated Yantai Yuhuangding Hospital of Qingdao University, Yantai, China; ^9^Department of Gynecology Oncology, Harbin Medical University Cancer Hospital, Harbin, China; ^10^Department of Obstetrics and Gynecology, Shengjing Hospital Affiliated to China Medical University, Shenyang, China; ^11^Department of Gynecology, Peking University People's Hospital, Beijing, China; ^12^Division of Life Sciences and Medicine, The First Affiliated Hospital of USTC, University of Science and Technology of China, Baohe District, China; ^13^Department of Gynecologic Oncology, Guangxi Medical University Cancer Hospital, Nanning, China; ^14^Department of Gynecology, The First Affiliated Hospital of Nanjing Medical University, Nanjing, China; ^15^The Third Hospital of Peking University, Beijing, China; ^16^Department of Gynecology and Obstetrics, The First Affiliated Hospital of Zhengzhou University, Zhengzhou, China; ^17^Department of Gynecology and Obstetrics, Tianjin Medical University General Hospital, Tianjin, China; ^18^Department of Gynecology and Obstetrics, Xinhua Hospital, Shanghai Jiaotong University School of Medicine, Shanghai, China; ^19^Department of Gynecology, People's Hospital of Xinjiang Uygur Autonomous Region, Urumqi, China; ^20^Department of Gynecology and Obstetrics, Development and Related Disease of Women and Children Key Laboratory of Sichuan Province, Key Laboratory of Birth Defects and Related Diseases of Women and Children, Ministry of Education, West China Second Hospital, Sichuan University, Chengdu, China; ^21^Department of Gynecology and Obstetrics, The Third Affiliated Hospital, Sun Yat-sen University, Guangzhou, China; ^22^Department of Gynecology, The Second Hospital of Shandong University, Jinan, China; ^23^Department of Gynecologic Oncology, Tianjin Central Hospital of Gynecology and Obstetrics, Affiliated Hospital of Nankai University, Tianjin, China; ^24^Department of Gynecologic Oncology, Tianjin Clinical Research Center for Gynecology and Obstetrics, Branch National Clinical Research Center for Gynecology and Obstetrics, Tianjin, China; ^25^Department of Obstetrics and Gynecology, Xiangya Third Hospital, Central South University, Changsha, China

**Keywords:** endometrial cancer, recurrence pattern, early recurrence, clinical features, risk factors

## Abstract

**Objective:**

The aim of the present study was to determine overall survival (OS) and risk factors associated with early recurrence in patients with FIGO I–II stage endometrial carcinoma (EC).

**Methods:**

Clinical features were retrospectively extracted from the database of China Endometrial Cancer Consortium from January 2000 to December 2019. A total of 2,974 patients with Federation International of Gynecology and Obstetrics (FIGO) I–II stage endometrial cancer were included. Kaplan-Meier survival analysis was used to assess OS and disease-specific survival. Cox proportional hazard model and Fine-Gray model were used to determine the factors related to OS. Binary logistic regression model was used to determine independent predictors of early relapse patients.

**Results:**

Of these 2,974 ECs, 189 patients were confirmed to have relapse. The 5-year OS was significantly different between the recurrence and non-recurrence patients (*p* < 0.001). Three quarters of the relapse patients were reported in 36 months. The 5-year OS for early recurrence patients was shorter than late recurrence [relapse beyond 36 months, *p* < 0.001]. The grade 3 [odds ratio (OR) = 1.55, 95%CI 1.17–2.05, *p* = 0.002], lymphatic vascular infiltration (LVSI; OR = 3.36; 95%CI 1.50–7.54, *p* = 0.003), and myometrial infiltration (OR = 2.07, 95%CI 1.17—3.65, *p* = 0.012) were independent risk factors of early relapse. The protective factor of that is progesterone receptor (PR)-positive (OR = 0.50, 95%CI 0.27–0.92, *p* = 0.02). Bilateral ovariectomy could reduce recurrence risk rate (OR = 0.26, 95%CI 0.14–0.51, *p* < 0.001).

**Conclusion:**

The OS of early relapse EC is worse. Grade 3, LVSI, and myometrial infiltration are independent risk factors for early relapse EC. In addition, the protective factor is PR-positive for those people and bilateral salpingo-oophorectomy could reduce the risk of recurrence.

## Highlights

- Early relapse FIGO I–II endometrial cancer patients had worse survival.
- Histological grade, LVSI, more than half of depth of myometrial infiltration, PR-negative are independent risk factors for early relapse EC. And bilateral salpingo-oophorectomy could reduce the risk of recurrence.

## Introduction

Endometrial cancer (EC) is one of the common malignant tumors of the female reproductive system (1). Eighty percent of ECs confined to the uterus and the prognosis is good (2). In recent years, studies have shown that the risk of early EC recurrence and death is increasing (3). The recurrence and 5-year overall survival (OS) rates of patients with FIGO I–II are 2–15 and 74–91%, respectively (4–6). Although many studies have pointed out that age, FIGO stage, pathological type, histological grade, depth of myometrial invasion, lymphatic invasion, and estrogen receptor (ER)-negative are risk factors for EC recurrence (7–9), there are still 218,000 patients who die from the disease every year in China (10, 11). EC is the most common gynecological malignancy in developed countries (12). In the United States, EC is the fourth most common cancer affecting women and the sixth most common cancer in terms of mortality (13). In addition, patients with FIGO stage I EC is grade 3, and women with stage II EC are generally considered to have highrisk early disease, but there is no clear definition of the best treatment (14, 15).

Although EC FIGO Stage I–II lesions are limited to the uterus, the recurrence rate and the risk of death from the disease are much lower than those of patients with FIGO III–IV EC, but this group of patients with early EC did experience recurrence. The shorter the recurrence time, the higher the risk of dying from the disease (16). The current research on recurrence factors is mainly focused on all patients with endometrial cancer. However, so far, the risk factors related to early recurrence in FIGO I–II has not been clearly identified. Therefore, the purpose of this study is to determine the clinical and pathological factors that predict the early recurrence of FIGO stage I–II EC and to improve the OS of those patients.

## Methods

### Patients

The information of the patients comes from the Academic Center of China Endometrial Cancer Association, which includes 30 academic centers from different regions of China. The research was approved by the Academic Center of China Endometrial Cancer Association to release these clinical data. We investigated the patients who underwent the surgery treatment and were diagnosed EC from January 2000 to December 2019. All women included in this study were diagnosed with FIGO stage Ia, Ib, or II and had follow-up data after the initial treatment.

### Clinical Information

Patient data was extracted from 30 institutions that maintain EC databases. For cases diagnosed as FIGO stage Ia, Ib, or II, we collected the following information from medical records: date of diagnosis, age at diagnosis, body mass index (BMI), LVSI, depth of myometrial infiltration, grade, histological type, estrogen/progesterone receptor, tumor surgery treatment, adjuvant treatment methods, follow-up time, recurrence time (as a continuous variable or dichotomous variable; 36 or >36 months), recurrence location, treatment after the relapse, survival period, and other cancer-related information. We collected data from various centers through standardized forms. Tumor surgery for patients with endometrial cancer is performed by a professional obstetrician and gynecologist. All surgical specimens were examined and interpreted by a gynecological pathologist in the hospital. The classification and stag of tumor structure adopt the 2009 FIGO standard. Patients were followed up every 3 months for the first 2 years, every 6 months for the next 3 years, and then once a year. After the initial treatment, physical examination, ultrasound examination, MRI, CT, or positron emission tomography (PET-CT) imaging examination confirmed that the tumor recurred as a recurrence.

### Subgroup Analyses

According to the time of recurrence, this study divided patients with FIGO stage I–II EC into early and late recurrence. Early recurrence was defined as the relapse time within 36 months after the patient received the initial treatment. Late relapse refers to a patient who relapses more than 36 months after the first treatment. Recurrence is categorized according to the location of recurrence as follows: (1) vaginal recurrence: the lesion appears in the vagina fornix; (2) pelvic recurrence: the lesion appears in the pelvic cavity, which is defined as a local recurrence. Distant recurrence is when the disease appears outside the pelvis (lung, liver, brain, and bone), but does not involve the vagina and pelvis.

We used binary logistic regression model and conditional reverse analysis to evaluate and analyze BMI, histopathology type, histological grade, depth of myometrial invasion, LVSI, estrogen receptor (ER), progesterone receptor (PR), tumor surgery treatment, and adjuvant treatment methods for its potential impact of the early relapse of patients with FIGO stage I–II. Kaplan Meier analyzed the OS and disease-specific survival (DSS) of patients with early recurrence and late relapse patients.

### Statistical Analysis

In univariate and multivariate analysis, we assessed the potential impact of age, BMI, LVSI, depth of muscle invasion, histopathology type, histological grade, ER, PR, treatment methods, relapse date, and recurrence site of the OS of patients with early EC. The Cox proportional hazard regression model and the Fine and Gray model determine factors related to OS after recurrence and report the hazard ratios (HR). Binary logistic regression model and conditional backward method were used to evaluate the risk factors of early relapsed patients. The characteristics of FIGO Ia, Ib, and II relapsed and non-relapsed patients were compared by Student's t-test, Chi-square test, and Fisher's Exact test. The group of early and late relapsed patients was also compared by those methods. Survival time refers to the date from the date of diagnosis to the date of death from any cause. The Kaplan-Meier refined algorithm was used to calculate OS and DSS. Log-rank test is also used to evaluate the survival difference curve. A *p* < 0.05 was considered significant. We performed all analyses using SPSS 26.0 (SPSS Inc., Chicago, IL, USA) and the R version 4.0.3.

## Results

### Patient Characteristics and Recurrence

**Figure 1:**
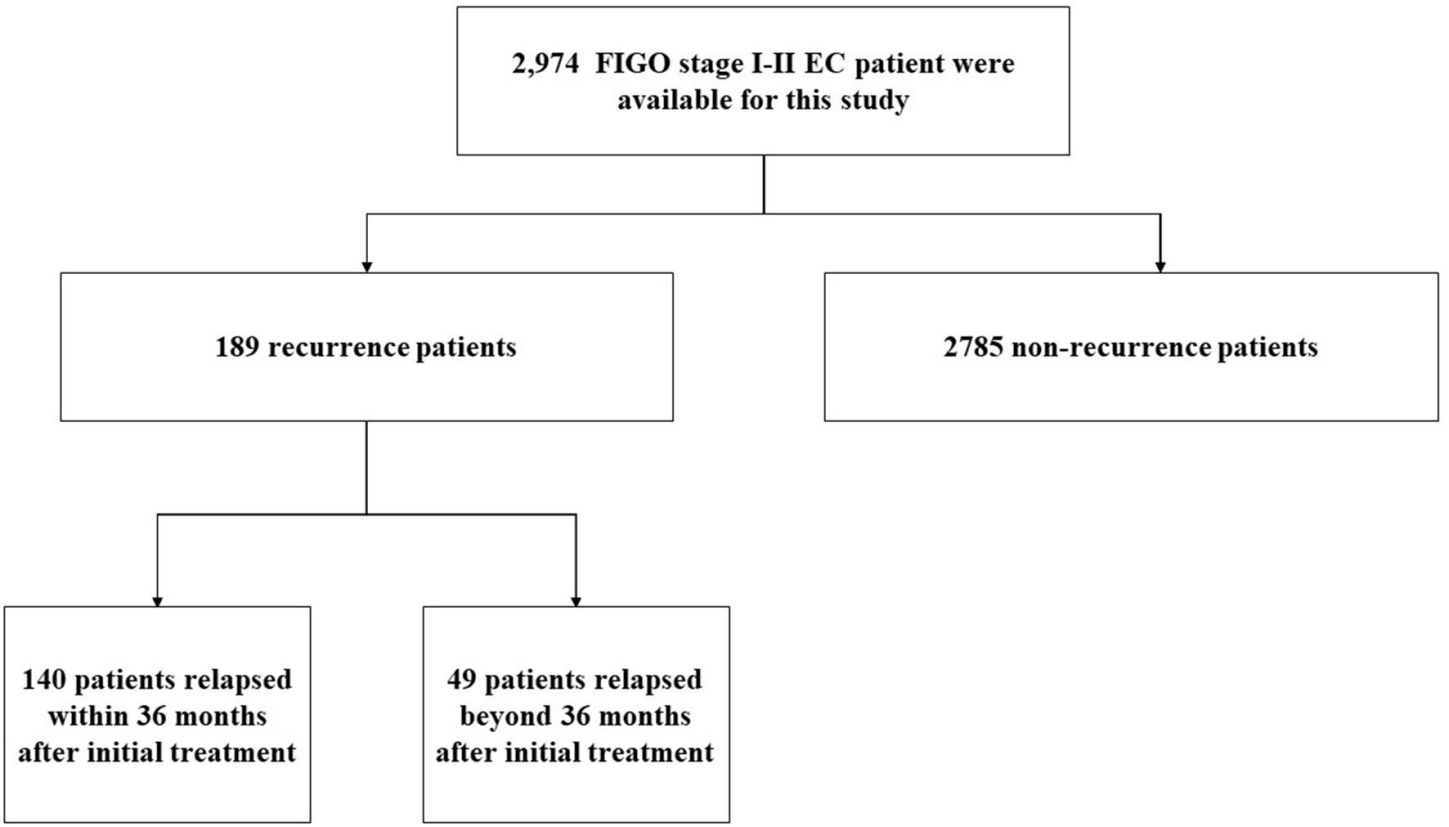
Flowchart of this study.

**Figure 2:**
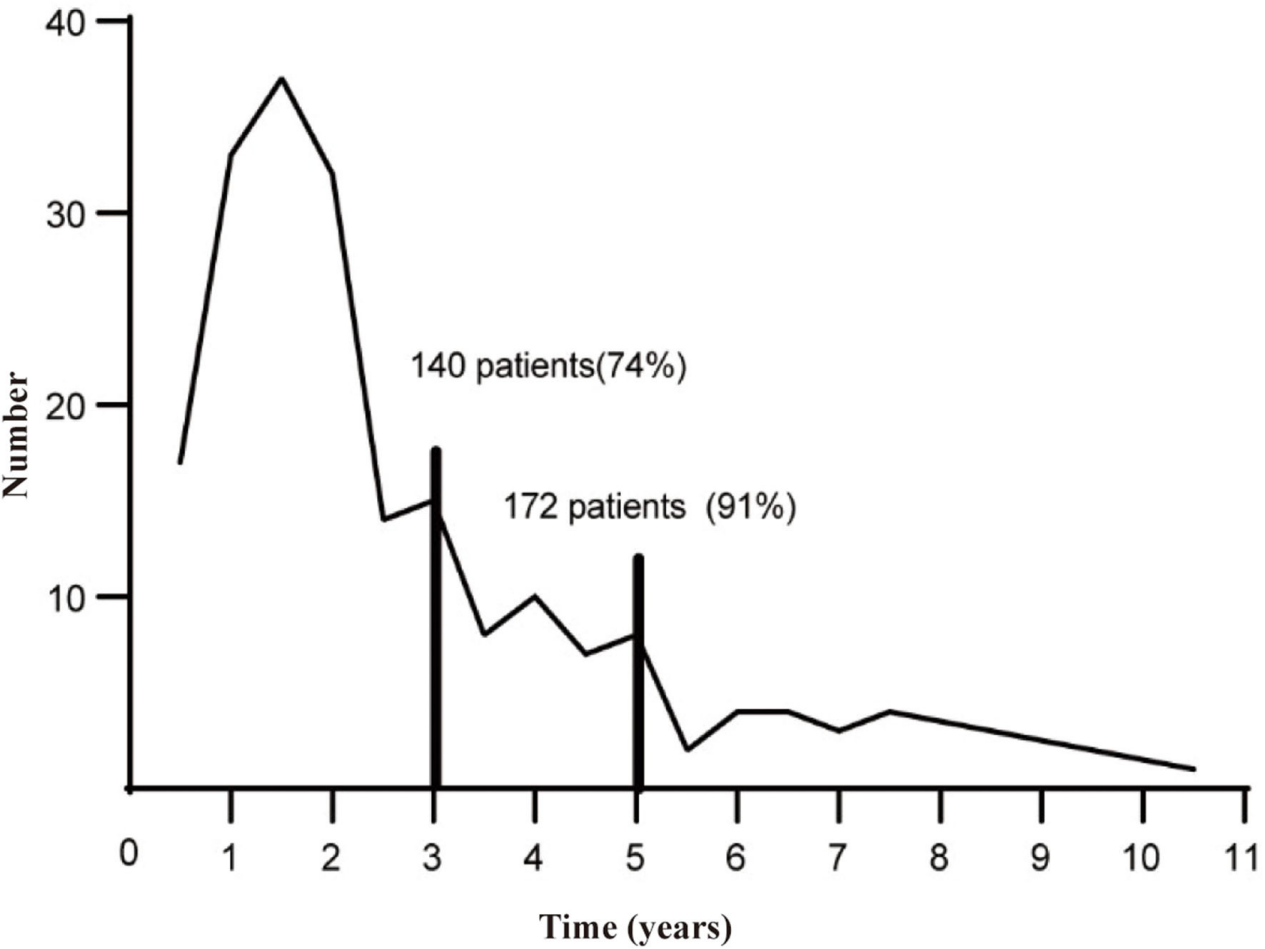
The time chart for patients first recurrence.

From January 2000 to December 2019, a total of 2,974 cases were diagnosed with FIGO stage I–II endometrial cancer, and the medical history and follow-up data were relatively complete in the China Endometrial Cancer Alliance (Figure 1). The median follow-up time of patients were 6.6 years. Among the 2,974 patients, 189 (6.4%) women had relapses (Table 1). The median time to relapse was 20.9 months, and the average age was 57.77 ± 10.36. There were 140 patients (74.0%) who relapsed within 36 months after the initial treatment, with an average age of 58.11 ± 10.17, which was older than the average age of patients who relapsed 36 months later (Figure 2; Table 1). The early recurrence rate of patients with non-endometrioid type was 18.6%. In other characteristics, early recurrence patients with G3, muscular invasion depth >1/2, LVSI positive, and ER- and PR-negative were 38.8%, 33.6%, 11.9%, 23.1%, and 30.8% respectively. This proportion was higher in late recurrence patients. There were 359 patients retained their ovaries, and the rest received bilateral ovariectomy. Then 65 of those who retained their ovaries experienced recurrence. And 947 patients received chemotherapy or radiotherapy. Among them, 88 patients with EC have relapsed.

**Table 1:** Baseline patient characteristics.

			**Recurrence (*****n*** **=** **189)**			***p***
	**Total (*n* = 2,974)**	**Non-recurrence (*n* = 2,785)**	**Total (*n* = 189)**	**≤36 m recurrence (*n* = 140)**	**>36 m recurrence (*n* = 49)**	
Age (y)	54.50 ± 9.53	54.28 ± 9.43	57.77 ± 10.36	58.11 ± 10.17	56.78 ± 10.96	0.441
**BMI (kg/m** ^**2**^ **)**
≤ 25	695	635 (55.3%)	60 (53.6%)	52 (55.3%)	8 (44.4%)	0.397
> 25	565	513 (44.7%)	52 (46.4%)	42 (44.7%)	10 (55.6%)	
Not report	1,714					
**Histology**
Endometrioid	2,765	2,606 (93.6%)	159 (84.1%)	114 (81.4%)	45 (91.8%)	0.086
Non-endometrioid	209	179 (6.4%)	30 (15.9%)	26 (18.6%)	4 (8.2%)	
**Grade**
G1–2	2,478	2,361 (84.8%)	117 (65.0%)	82 (61.2%)	35 (76.1%)	0.068
G3	487	424 (15.2%)	63 (35.0%)	52 (38.8%)	11 (23.9%)	
Not report	9					
**Myometrial invasion**	
≤ 1/2	2,231	2,124 (85.5%)	107 (67.7%)	79 (66.4%)	28 (71.8%)	0.531
> 1/2	410	359 (14.5%)	51 (32.3%)	40 (33.6%)	11 (28.2%)	
Not report	333					
**LVSI**						
Positive	71	55 (5.7%)	16 (22.5%)	13 (11.9%)	3 (8.1%)	0.762
Negative	2,262	2,132 (94.3%)	130 (77.5%)	96 (88.1%)	34 (91.9%)	
Not report	641					
**ER**						
Positive	1,404	1,332 (89.0%)	72 (80%)	50 (76.9%)	22 (88.0%)	0.239
Negative	182	164 (11.0%)	18 (20%)	15 (23.1%)	3 (12.0%)	
Not report	1,388					
**PR**						
Positive	1,385	1,319 (88.3%)	66 (73.3%)	45 (69.2%)	21 (84%)	0.156
Negative	199	175 (11.7%)	24 (26.7%)	20 (30.8%)	4 (16%)	
Not report	1,390					
**Treatment before recurrence**
**Bilateral oophorectomy**						
No	359	294 (10.6%)	65 (34.8%)	49 (35%)	16 (34.1%)	0.905
Yes	2,611	2,489 (89.4%)	122 (65.2%)	91 (65%)	31 (65.9%)	
Not report	4					
**Chemotherapy**
No	2,168	2,043 (73.4%)	125 (66.1%)	92 (65.7%)	33 (67.3%)	0.863
Yes	806	742 (26.6%)	64 (33.9%)	48 (34.3%)	16 (32.7%)	
**Radiotherapy**	
No	2,833	2,668 (95.8%)	165 (87.3%)	121 (86.4%)	44 (89.8%)	0.627
Yes	141	117 (4.2%)	24 (12.7%)	19 (13.6%)	5 (10.2%)	
**Treatment after recurrence**
**Chemotherapy**
No	68		68 (48.9%)	49 (51.0%)	19 (51.4%)	0.348
Yes	65		65 (51.1%)	47 (49.0%)	18 (48.6%)	
Not report	56					
**Radiotherapy**
No	108		108 (71.1%)	82 (71.3%)	26 (70.3%)	1.000
Yes	44		44 (28.9%)	33 (28.7%)	11 (29.7%)	
Not report	37					
**Recurrence site**
Distant	94		94 (49.7%)	71 (50.7%)	23 (46.9%)	0.740
Local	95		95 (50.3%)	69 (49.3%)	26 (53.1%)	
**Median time to recurrence (m)**			20.90 (11.68, 38.45)	15.92 (8.97, 22.73)	56.43 (44.28, 71.60)	
**Median time to OS (m)**	79.06 (66.06, 109.08)		38.17 (22.88, 54.20)	29.08 (17.37, 43.78)	61.60 (48.42, 80.78)	

### Factors Related to Overall Survival and Recurrence

In the univariable analysis, ER, recurrence site, and recurrence time were associated with the OS of patients whose cancer had recurred (Table 2). Multi-factor competitive Fine and Gray model analysis of the clinical and pathological characteristics revealed that there are two factors that are significantly related to the OS of relapsed patients. They are ER (SHR = 0.23, 95%CI 0.08–0.63, *p* = 0.004) and recurrence time (SHR = 2.58, 95%CI 1.03–6.44, *p* = 0.042) (Table 3). ER-negative patients and patients who relapse within 36 months after initial treatment have a higher risk of dying from endometrial cancer than patients who are ER-positive and whose recurrence time is more than 36 months.

**Table 2:** Univariate analysis on post-relapse overall survival time.

	**HR**	**95%CI**		***P***
**BMI**				
≤ 25	Reference			
> 25	1.23	0.62	2.45	0.56
**Histology**				
Non-endometrioid	Reference			
Endometrioid	0.93	0.56	1.53	0.76
**Grade**				
G1-2	Reference			
G3	1.18	0.86	1.62	0.31
**Myometrial invasion**				
≤ 1/2	Reference			
> 1/2	1.34	0.71	2.50	0.36
**LVSI**				
Negative	Reference			
Positive	0.97	0.35	2.74	0.96
**ER**				
Negative	Reference			
Positive	0.18	0.08	0.44	<0.001
**PR**				
Negative	Reference			
Positive	0.56	0.23	1.39	0.21
**Bilateral oophorectomy**				
No	Reference			
Yes	0.98	0.54	1.79	0.96
**Recurrence site**				
Distant	Reference			
Local	0.47	0.23	0.93	0.03
**Recurrence time**				
> 36	Reference			
≤ 36	3.09	1.65	5.78	<0.001

**Table 3:** Multivariate Fine-Gray competing risk regression analysis on postrelapse overall survival time.

	**SHR**	**95%CI**		***P***
**ER**				
Negative	Reference			
Positive	0.23	0.08	0.63	0.004
**Recurrence site**				
Distant	Reference			
Local	0.56	0.17	1.84	0.34
**Recurrence time**				
> 36	Reference			0.042
≤ 36	2.58	1.03	6.44	

### Recurrence Overall Survival and Disease-Specific Survival

**Figure 3:**
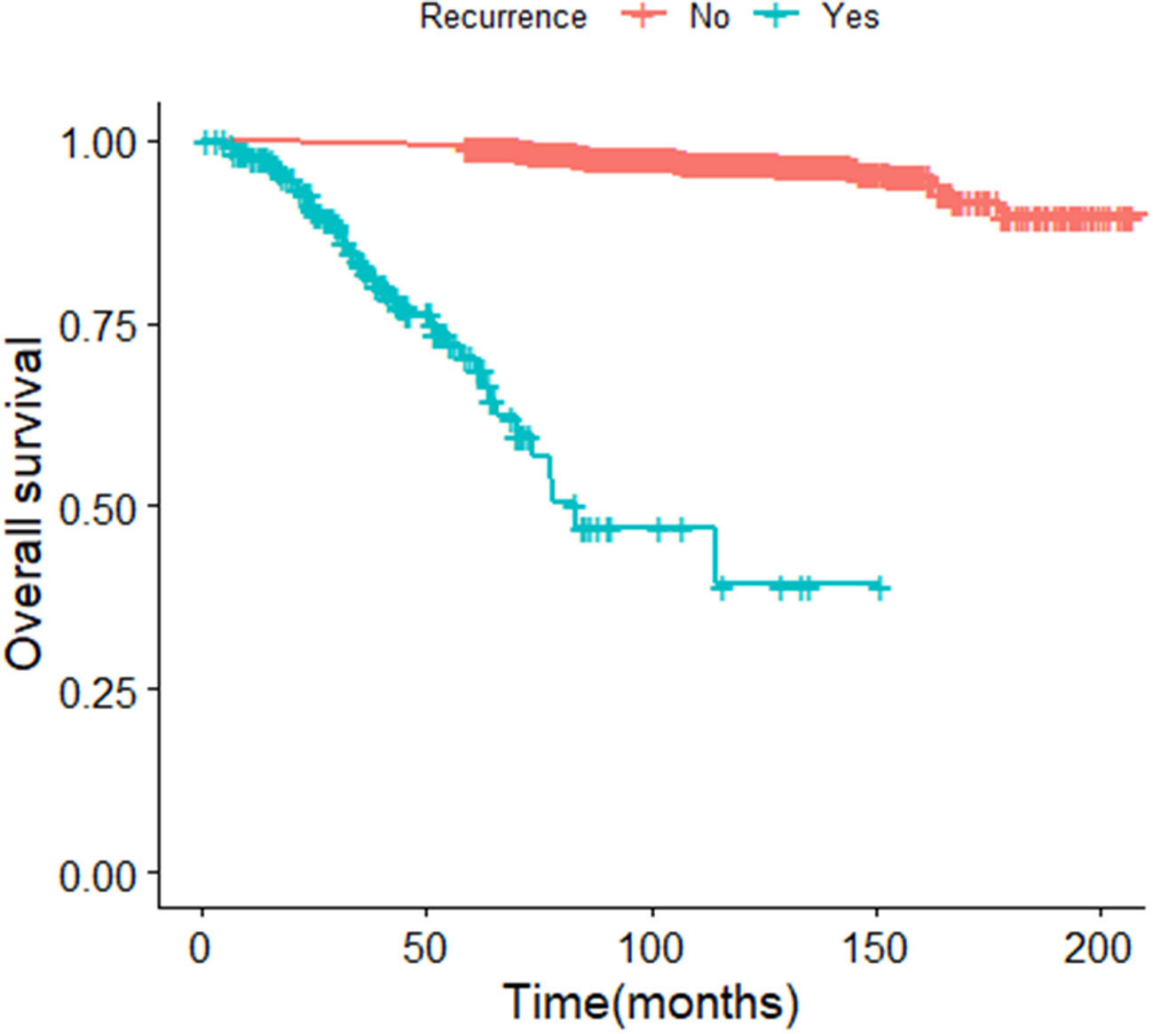
Kaplan-Meier curve of overall survival for early EC patients (*p* < 0.001).

**Figure 4:**
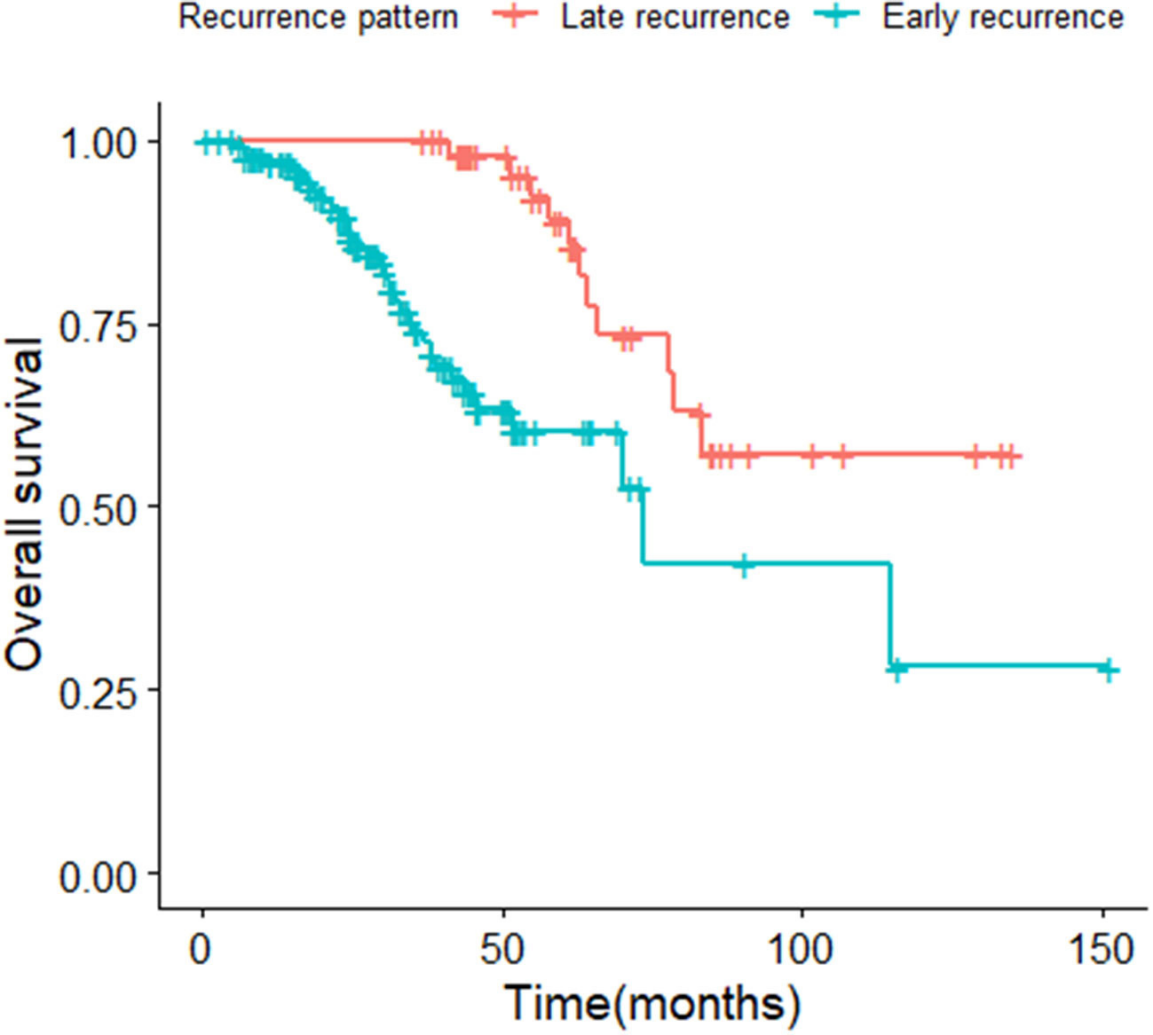
Kaplan-Meier curve of overall survival for patients with early and late relapse (*p* < 0.001).

**Figure 5:**
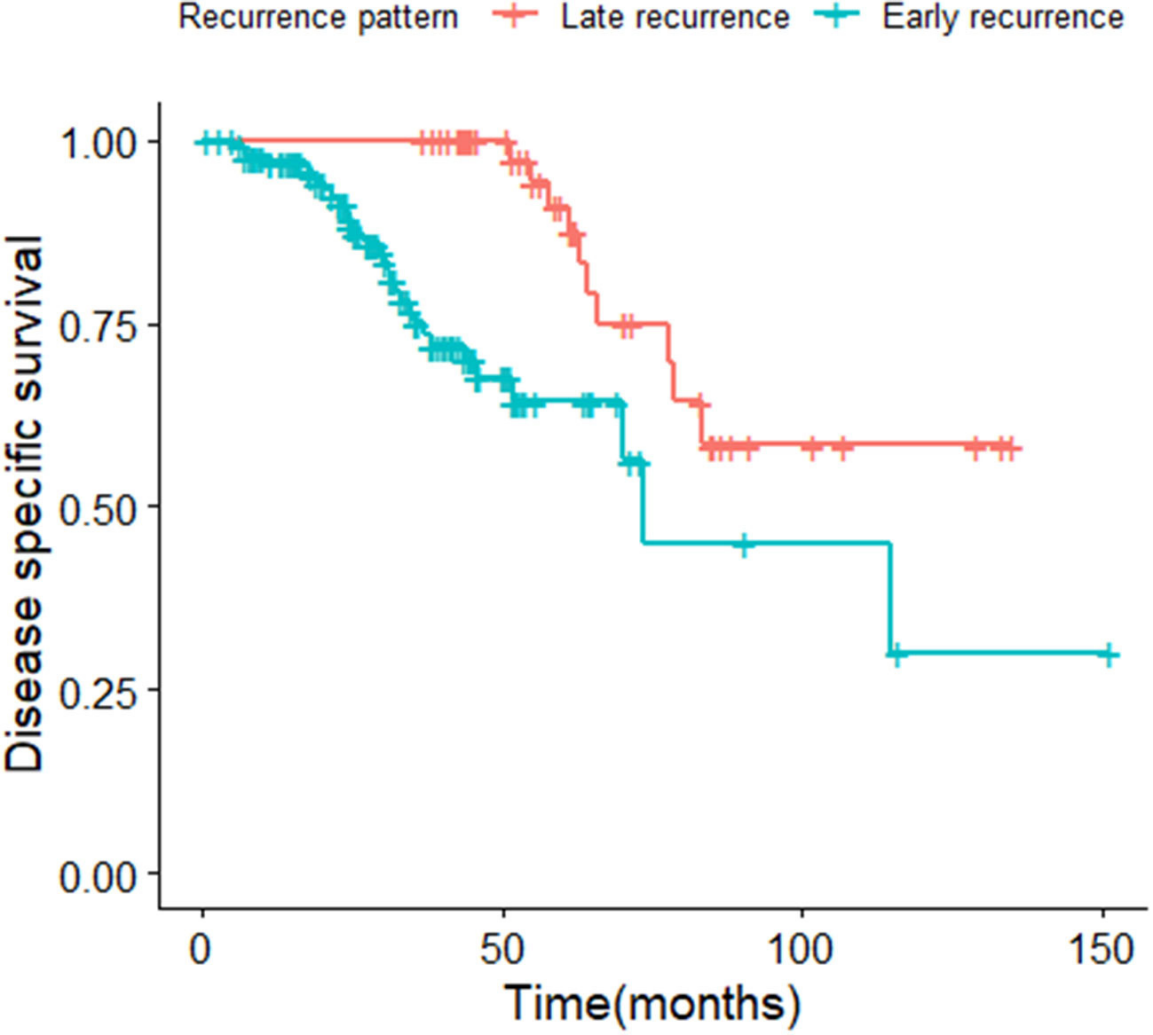
Kaplan-Meier curve of disease specific survival for patients with early and late relapse (*p* = 0.008).

The median survival time of the included study population was 79.06 months. Patients who relapsed after the initial treatment had a lower survival rate than those who did not relapse (*p* < 0.001, Figure 3). The median time between surgery and recurrence was 20.90 months. Of these, 140 had a relapse within 36 months. The 5 year OS of these patients was lower than the patients who relapsed 36 months later (*p* < 0.001; Figure 4). In addition, the 5-year DSS of patients with early relapse is lower than the patients with late relapse (*p* = 0.008; Figure 5).

### Factors Related to Relapse in Short Periods

The single-factor and multi-factor binary logistic regression model and conditional regression analysis of the clinical and pathological risk factors of the patients found that there are five factors related to the early relapse patients with EC. The independent risk factors are grade (OR = 1.55, 95%CI 1.17–2.05, *p* = 0.002), myometrial invasion (OR = 2.07, 95%CI 1.17–3.65, *p* = 0.012), and LVSI (OR = 3.36, 95%CI 1.50–7.54, *p* = 0.003). The protective factor of that is PR-positive (OR = 0.50, 95%CI 0.27–0.92; *p* = 0.02). Bilateral ovariectomy could reduce recurrence risk rate (OR = 0.26, 95% CI 0.14–0.51, *p* < 0.001) (Table 4). A total of 141 patients in early EC received radiotherapy, of which 59 were patients with FIGO stage Ia, and the remaining patients were FIGO stages Ib and II. The number of patients receiving radiotherapy recurred was 24, of which 58% patients was FIGO stages Ib and II (Supplementary Table 1).

**Table 4:** Logistics regression model analysis for early recurrence patients.

	**Univariate analysis**				**Multivariate analysis**			
	**OR**	**95%CI**		***P***	**OR**	**95%CI**		***P***
**BMI**								
≤ 25	Reference							
> 25	0.99	0.65	1.52	0.97				
**Histology**								
Non-endometrioid	Reference				Reference			
Endometrioid	0.30	0.19	0.48	<0.001	1.41	0.48	4.14	0.53
**Grade**								
G1-2	Reference				Reference			
G3	1.87	1.56	2.24	<0.001	1.55	1.17	2.05	0.002
**Myometrial invasion**								
≤ 1/2	Reference				Reference			
> 1/2	2.95	1.98	4.38	<0.001	2.07	1.17	3.65	0.012
**LVSI**								
Negative	Reference				Reference			
Positive	4.77	2.66	8.56	<0.001	3.36	1.50	7.54	0.003
**ER**								
Negative	Reference				Reference			
Positive	0.44	0.23	0.75	0.004	0.79	0.33	1.87	0.588
**PR**								
Negative	Reference				Reference			
Positive	0.30	0.17	0.52	<0.001	0.50	0.27	0.92	0.02
**Bilateral oophorectomy**								
No	Reference				Reference			
Yes	0.22	0.16	0.31	<0.001	0.26	0.14	0.51	<0.001
**Chemotherapy**								
No	Reference							
Yes	1.43	0.99	2.05	0.051				
**Radiotherapy**								
No	Reference				Reference			
Yes	3.49	2.08	5.85	<0.001	2.60	1.17	5.82	0.02

## Discussion

From January 2000 to December 2019, a total of 2,974 patients with FIGO stage I–II EC were used for analysis in this study. We identified 189 patients with FIGO stage I–II EC who relapsed.

At present, the risk factors of the EC recurrence include age, FIGO stage, pathological type, histological grade, BMI, LVSI, depth of myometrial invasion, ER, and PR-negativity (17–19). Presently, the clinical treatment for EC is mainly decided based on histopathological characteristics. The patient with EC is followed up every 3 months during the first 3 years, every 6 months during the next 2 years, and annually thereafter. Early EC that is confined to the uterus generally had better prognoses. However, both the recurrence incidence and fatality rates of these patients have increased consistently in recent years (3). The recurrence rate of EC FIGO stage I–II observed in this study and other studies ranged from 5 to 15% (20, 21). The 5-year OS of relapse patients was significantly shorter than that of non-recurrence patients (*p* < 0.001). According to the analysis of competitive risk model, recurrence time and ER-negative are risk factors affecting OS. Among patients with FIGO stage I–II EC, three quarters of them relapsed within 3 years, consistent with another research (22). At present, the research on risk factors for EC recurrence mostly focuses on FIGO stage and recurrence site. The risk factors for EC recurrence in the short term are not clear. Therefore, this article divides patients into early recurrence and late recurrence according to the time of recurrence.

The earlier relapse of EC occurs, the greater the subsequent mortality risk (16). The 5-year OS of early relapse patients with EC is 60.3%, whilst the OS of late relapse women are closer to 81%. PR-positivity is a protective factor for early relapse patients with EC. Bilateral salpingo-oophorectomy could reduce the risk of short-term recurrence. In addition, depth of myometrial invasion, LVSI, and a Grade of 3 were found to be independent risk factors for those recurrence patients, which are consistent with the results of many current reports (19, 21). In addition, some studies showed that obesity is an important risk factor for patients with EC (23, 24). Although in this research, we found no evidence to support this. The reason might be that our observation mainly covered patients with FIGO stage I–II EC.

The immunohistochemical expression of ER and PR has a good correlation between curettage and final hysterectomy specimens (25, 26). In a prospective multicenter trial, the lack of hormone receptor in endometrial cancer is associated with a reduced survival rate and lymph node metastasis (27). Therefore, a number of follow-ups within 3 years after surgery for patients who are PR-negative and had more than half of depth of myometrial invasion and grade 3 is recommended for early diagnosis in order to improve OS.

Generally, ovaries should be better preserved in young women with low-grade and early EC (28). This is because when women are premenopausal, bilateral oophorectomy causes an immediate onset of menopause with large reduction of ovarian hormone level. These patients would not only experience menopausal symptoms that endanger the quality of life, but also metabolic disorders of female hormone levels, which are prone to develop rarefaction of bone or autonomic nerve dysfunction. In addition, lack of hormone would increase the risk of future cardiovascular disease. However, according to our study, it is not recommended to keep the ovaries in patients who are PR-negative, are with more than half of depth of myometrial invasion or with G3.

In addition, we found that early-stage patients receiving radiotherapy and chemotherapy did not improve OS, and that radiotherapy may be an independent risk factor for short-term recurrence. This may be caused by 58.2% of patients with FIGO stage Ib and II receiving radiotherapy. The overall OS of patients with FIGO stage Ia is better than that of patients with FIGO stage Ib and II. At present, several randomized clinical studies have been conducted for adjuvant therapy. Using adjuvant therapy in women with advanced EC could improve OS (29). According to a clinical randomized phase III trial of advanced EC adjuvant therapy, the combination of doxorubicin-cisplatin chemotherapy has a significant improvement in OS compared with whole-abdominal irradiation (HR = 0.68; 95%CI = 0.52–0.89; *p* < 0.01) (30). On the other hand, there is no clear evidence that proved the survival advantage of adjuvant therapy for stage I and II disease (20). For patients with EC with low risk, adjuvant therapy is not recommended because of the low risk of recurrence. For middle-risk patients with EC patients among FIGO stage I–II, although radiotherapy could reduce the local recurrence risk of the patient, it does not improve the OS of the patient (3, 31). According to a randomized clinical trial comparing adjuvant radiotherapy and chemotherapy, it is found that patients with early-stage EC receiving radiotherapy or chemotherapy did not improve OS (30, 32). Whether our result would be applicable to patients with FIGO stage I–II who received radiotherapy needs to be further investigated.

This study also has some limitations. First of all, this study is a retrospective study, and the bias cannot be ruled out. In addition, these cases come from multiple hospital centers, and each center has specific differences in the evaluation and treatment of patients. However, considering the overall prognosis of endometrial cancer patients, in order to obtain a sufficient sample size, so many medical centers are included. On the other hand, due to the limitation of retrospective research, this article did not include molecular typing, and it is impossible to analyze the causes of EC recurrence in the short term from the perspective of molecular characteristics. This needs to be improved by subsequent research. Despite the above-mentioned limitations, this study helps to understand the influencing factors of EC recurrence in the short term and provides a reference basis for the treatment and management of patients with EC in emergency underdeveloped areas or primary hospitals that lack molecular diagnostic methods.

In conclusion, this study clarified the prognosis and recurrence factors of FIGO I–II EC. The OS of patients with early recurrence is much lower than that of patients with late recurrence. More than half of depth of myometrial invasion, LVSI, and histological grade 3 are independent risk factors for short-term recurrence. PR-positivity is a protective factor for short term recurrence of patients with EC. Lastly, Bilateral salpingo-oophorectomy could reduce the risk of the early recurrence.

## Data Availability Statement

The raw data supporting the conclusions of this article will be made available by the authors, without undue reservation.

## Ethics Statement

The study was approved by the Academic Center of China Endometrial Cancer Association. The Local Institutional Review Board does not require ethical approval, as this is a use of routinely collected data, and therefore no written informed consent was obtained.

## Author Contributions

YD and KS contributed to writing the original draft. GC and CS contributed to the review and editing. YS, CZ, SY, CX, MX, GL, JihL, BL, JW, WZ, JZ, WC, HG, RG, FX, XW, LH, BW, and YF contributed to the data collection. XZ, XL, PZ, JZ, JM, WL, XY, ZW, JinL, YF, KL, XC, and JJ contributed to the formal analysis. DL and BK: supervision. All authors have read and agreed to the published version of the manuscript.

## Funding

This work was supported by the Nature and Science Foundation of China (81874106 and 82073259), the Key R&D Program of Hubei Province (2020BCA067), and the Hubei Province Science Fund for Distinguished Young Scholars (2020CFA066).

## Conflict of Interest

The authors declare that the research was conducted in the absence of any commercial or financial relationships that could be construed as a potential conflict of interest.

## Publisher's Note

All claims expressed in this article are solely those of the authors and do not necessarily represent those of their affiliated organizations, or those of the publisher, the editors and the reviewers. Any product that may be evaluated in this article, or claim that may be made by its manufacturer, is not guaranteed or endorsed by the publisher.
