# Supplementary material for: Risk Factors and Prognosis of Early Recurrence in Stage I–II Endometrial Cancer: A Large-Scale, Multi-Center, and Retrospective Study
Source: Front Med (Lausanne). 2022 Apr 14;9:808037. doi: 10.3389/fmed.2022.808037 (PMC9046937; doi:10.3389/fmed.2022.808037)
Supplement: Supplementary file 1 [file Table_1.docx]

Supplementary Table 1 baseline of early EC patients received radiotherapy

|  | Total (n=141) | Recurrence (n=24) | Non-recurrence (n=117) |
| --- | --- | --- | --- |
| **FIGO** |  |  |  |
| **Ia** | 59 (41.8%) | 10 (41.7%) | 49 (41.8%) |
| **Ib-II** | 82 (58.2%) | 14 (58.3%) | 68 (58.2%) |
